# Surgical Management and Outcome Experience of 53 Cerebellopontine Angle Meningiomas

**DOI:** 10.7759/cureus.1538

**Published:** 2017-08-03

**Authors:** Xiaosheng He, Weiping Liu, Yangang Wang, Jun Zhang, Buqing Liang, Jason H Huang

**Affiliations:** 1 Neurosurgery, Xijing Hospital, Fourth Military Medical University, Xi'an, China; 2 Neurosurgery, Chinese Pla General Hospital, Beijing, China; 3 Neurosurgery, Baylor Scott & White Health

**Keywords:** meningioma, cerebellopontine angle, surgical resection, retrosigmoid approach

## Abstract

Background

Meningiomas follow schwannomas as the second most common cerebellopontine angle (CPA) tumors. We investigate the diagnosis, management, and prognosis of this disease.

Methods

We reviewed the cases with the CPA meningiomas in our institution in Shaanxi, China from January 2012 to December 2015. Charts were retrospectively examined and patients were divided into two groups: 1) surgical treatment with a retrosigmoid approach for tumor resection and 2) stereotactic radiosurgery treatment only. Patients were followed up and outpatient records were also reviewed.

Results

Forty-nine patients underwent surgical resection via the retrosigmoid approach, while the other four underwent Gamma Knife® stereotactic radiosurgery (Elekta AB, Stockholm, Sweden) only. The most common presenting symptoms included hearing loss/tinnitus, vertigo, and headache; only 8.2% were asymptomatic. The largest diameter and base of each tumor varied from 4.0 to 5.5 cm and 3.0 to 5.0 cm, respectively. The tumors extended into different directions, mainly towards the tentorium and internal acoustic meatus (IAM). Eighty-three percent of surgical patients had a gross total resection. One death occurred due to pulmonary inflammation. Tumor recurrence was noted in 6.1% of patients. Postoperative trigeminal disturbance, facial nerve palsy, and hearing deterioration or loss were the most common immediate and delayed postoperative complications; most patients partially or completely recovered after hospital discharge. Intraoperative neuro-electrophysiological monitoring, complete resection, and postoperative radiation were key factors for reducing complications and recurrence.

Conclusions

The retrosigmoid approach offers an ideal visual field for exposing and resecting CPA meningiomas in a large series of cases. In our experience, it is one of the most useful and commonly used surgical approaches for removing meningiomas of this region.

## Introduction

Cerebellopontine angle (CPA) meningiomas account for 1.5% of intracranial meningiomas and dominated (58.3%) in a series of meningiomas of the posterior skull base [1-2]. Of all CPA tumors, meningiomas are the second-most frequent tumor in this area [3]. They are benign tumors commonly seen in adults, which usually present after a long asymptomatic period.

Though full of challenges, surgical resection remains the main strategy to cope with this disease at present. Considering the slow growth of the tumor and the possible complications secondary to intraoperative insult and damage to vital anatomical structures, such as the cranial nerves, the decision on the necessity of complete surgical resection remains critical. What’s more, meningiomas occupying the same CPA region may have quite different origins. The relative locations of the meningiomas to the surrounding crucial organs determine whether surgical resection or conservative management is favored. Postoperatively, how additional non-surgical treatments assist in the prevention of recurrence is also important. As the knowledge related to the clinical anatomy of the skull base increases, more surgical approaches for CPA meningiomas have been developed and widely applied. Similar to other intracranial tumors, the indications for surgery alone or combining with adjuncts, such as radiation therapy, also need to be carefully balanced by the neurosurgical team. Without a doubt, these considerations will be universal to all intracranial meningiomas, and even other benign tumors.

This study analyzes the surgical results from a group of patients with initial radiographic diagnoses of CPA meningioma, confirmed by postoperative pathologies. A retrosigmoid approach was used in all surgical cases. Clinical parameters, such as symptoms/syndromes, radiographic characteristics, surgical strategies, and other comprehensive treatments, were also investigated to obtain insight into the factors that affect prognosis.

## Materials and methods

Patient population

**Figure 1:**
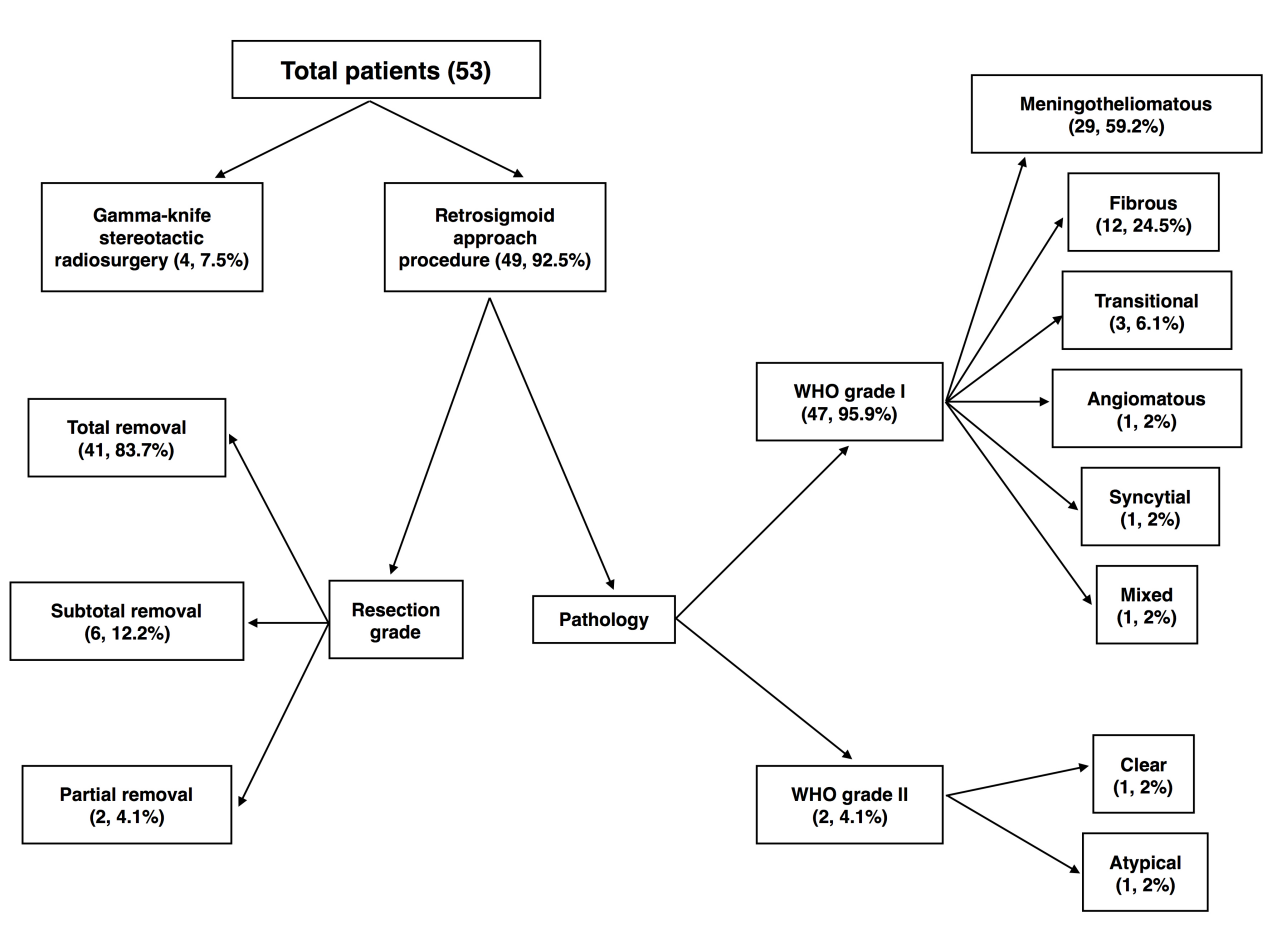
Resection Grade and Pathology of the Surgical Patients

This study was approved by the Ethics Committee of Xijing Hospital, Fourth Military Medical University. Patient consents were waived. All the radiographic imaging studies, including magnetic resonance imagings (MRIs), were reviewed by independent radiologists. Patients with bilateral meningiomas were excluded for the purity of the sample. A total of 53 patients, consecutively admitted to Xijing Hospital, Fourth Military Medical University from January 2012 to December 2015 with an initial radiographic diagnosis of CPA meningioma, were included in this study (Figure 1, Table 1).

**Table 1:** Patient Characteristics at the Time of Admission CT: computerized tomography; IAM: internal acoustic meatus; MRI: magnetic resonance imaging

The Clinical Characteristics of Patients Receiving Surgical Operation (n = 49)	
Sex	
% of female	57.1% 28/49
% of male	42.9% 21/49
Age	
Mean (yrs)	55.8
Range (yrs)	36-78
≥ 50 (yrs)	34
Enhanced MRI	
Largest diameter (cm)	4-5.5
Base width (cm)	3-5
Left (cases)	28
Right (cases)	21
CT window	
Dilated IAM (cases)/opening width (cm)	14/1-2
Reactive thickening of the petrous bone (cases)	32
Symptoms (cases)	
Hearing loss/tinnitus	21
Vertigo	20
Headache	19
Gait instability	4
Limb numbness/unableness	4
Facial pain/numbness	4
Nausea/vomiting	3
Facial palsy	3
Hemifacial spasm	1
Throat discomfort	1
Swallowing disorder	1
Ptosis of the eyelid	1
No obvious symptoms (cases)	3
Average duration (from symptoms complaint to hospital admission, months)	19.4

Surgical procedures

A retrosigmoid approach was used for all surgical cases. Each patient was placed in a lateral position with his/her head turned towards the opposite side of the tumor and fixed by Mayfield® framework (Integra LifeSciences Corp., Cincinnati, OH). An “S”-shaped scalp incision about 8 - 10 cm was made behind the ear. The incision was long enough to allow an ideal craniotomy to obtain a large bone flap. In order to get the largest exposure for the convenience of surgery, the mastoid air cells were opened and the sigmoid sinus was partially exposed. Sealing air cells with bone wax helped to avoid postoperative leakage of cerebrospinal fluid (CSF). The dura was incised in a radial fashion and the cerebellar hemisphere was gently retracted to expose and open the cerebellomedullary cistern. The CSF was released in a great degree to ensure a wider operative space. A serpentine retractor was used to get a stable operative window. Difficult exposures were encountered in three patients who had large tumors (> 5 cm) due to intraoperative cerebellar protrusion and the lateral one-third of the cerebellar hemispheres on the tumor side were removed. Ventricular puncture and CSF drainage were performed before craniotomy in two cases who demonstrated severe obstructive hydrocephalus and evidence of chronic foramen magnum herniation. Preoperatively, 26 cases were analyzed angiographically for the assistance of predicting tumor attachments. The tumors were debulked in a piecemeal fashion under an operative microscope. Coagulating and carbonizing the tumor tissues fruited bloodless resection. Ultrasonic aspiration (USA) was applied in 19 cases to ease the operative process when fragmentation, emulsification, and aspiration of soft and firm tumor tissue were desirable intraoperatively.

Neuroelectrophysiological monitoring

Neuroelectrophysiological monitoring (Epoch XP Neurological Workstation, Axon Systems, Hauppauge, NY) of the cranial nerves and brainstem functions included spontaneous electromyogram for cranial nerve V (trigeminal nerve, CN V), evoked electromyogram for facial nerve CN VII, spontaneous electromyogram for accessory nerve CN XI, and brainstem auditory evoked potential (BAEP). An alarm would notify the surgeons if these nerves and brainstem were disturbed during intraoperative retracting, bisecting, or tumor resection. If an alarm was triggered, the ongoing manipulation would be halted immediately until the neuroelectrophysiological monitoring confirmed the nerves to be stable.

Stereotactic radiosurgery

Gamma Knife® stereotactic radiosurgery (Elekta AB, Stockholm, Sweden) was utilized for either a treatment for small, asymptomatic CPA meningiomas or as a prevention of recurrence postoperatively. The radiation doses of 12-15 Gy were applied depending on the volumes of the target [4].

Data accumulation and statistical analysis

Detailed clinical materials were obtained from the clinical data bank in our hospital. The data, collected by complying with national laws on ethics, was summarized and processed using the Statistical Package for Social Sciences (SPSS) (IBM SPSS Statistics, Armonk, NY) software. Non-parametrical tests were performed with statistically significant differences defined as p value < 0.05.

## Results

Demographic information

Within this series of 53 cases, 49 underwent surgical resection and the other four were treated with stereotactic radiotherapy (Gamma Knife) based on the sizes of the tumors (< 2 cm diameter). The clinical characteristics of the population are listed in Table 1. Within the cases treated by surgical resection, 57.1% were female, the mean age was 55.8 years (ranged from 36 to 78 years) with a majority of ≥ 50 years (69.4%; 34/49), and the patients had illness courses ranging from two weeks to five years. The most common early symptoms of onset included hearing loss/tinnitus (21/53), vertigo (20/53), and headache (19/53) (Table 1). Three cases were diagnosed in routine physical examinations and presented with no obvious symptoms but growing tumors. The average duration from initial onset of symptoms to hospital admission was 19.4 months.

Tumor extension and origin

Besides dominating mainly the posterior aspect of the petrous part of the temporal bone, the meningiomas also extended into other directions, including the tentorium (32/53), internal acoustic meatus (IAM) (16/53), clivus (8/53), jugular foramen (3/53), and cavernous sinus (3/53). Ten cases involved tumors with extensions in two or more directions. Among all operated patients, enhanced magnetic resonance imaging (MRI) revealed the largest diameter and base varying from 4 to 5.5 cm and 3.0 to 5.0 cm, respectively. There were 28 cases with left CPA meningioma and 21 cases with right-sided CPA meningioma. Three patients presented with a meningioma with the largest diameter over 5 cm and obstructive hydrocephalus. According to computerized tomography (CT) window data, 14 patients had a dilated IAM with an opening of 1 to 2 cm, and 32 had reactive thickening of the petrous bone.

**Figure 2:**
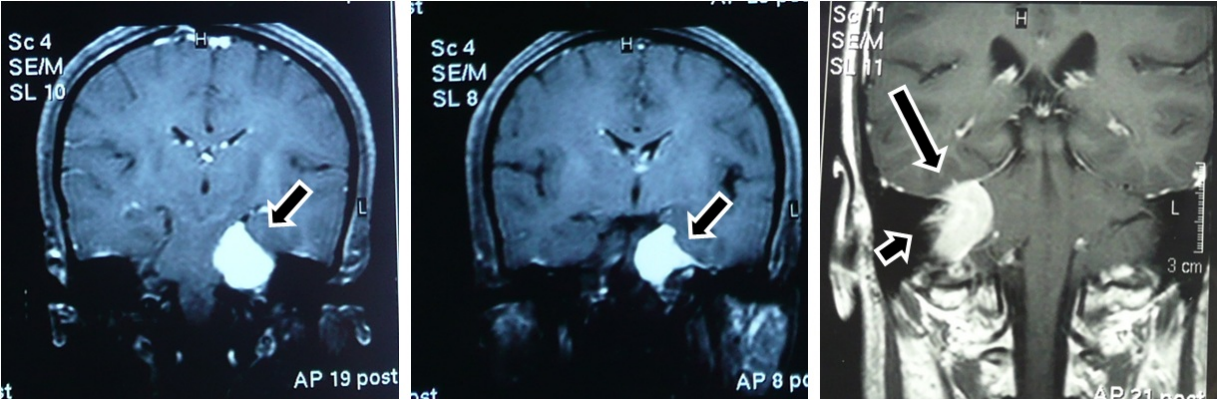
Preoperative MRI with Contrast Demonstrating the Origins and Extensions of Cerebellopontine Angle Meningiomas (A) Tumor with a wide base origin at the tentorium cerebelli (arrow) and an extension to the tentorium cerebelli hiatus compressing the pons. (B) The rodent-tailed sign of this tumor (arrow) revealed a larger origin from the tentorium and petrous bone. (C) The coarse base indicated that the tumor originated largely at the back of petrous bone (arrow) and partially at the tentorium cerebelli (arrowhead). MRI: magnetic resonance imaging

Three types of relationships were observed between the tumor and dura: attached but not adhered, tightly adhered but easily separated, and invading with impaired dura. The last was considered to indicate the origin of the meningioma. In this surgical group, the tumor originated from the petrous part of the temporal bone in 42 patients (39 only at the posterior surface, three also around the petrous tip), on the IAM in three, and on the cerebellar tentorium in four (two simultaneously coming from the wall of the sigmoid sinus). Preoperative radiographic examination served as an invaluable guide in the intraoperative identification of the tumor origin (Figure 2).

**Figure 3:**
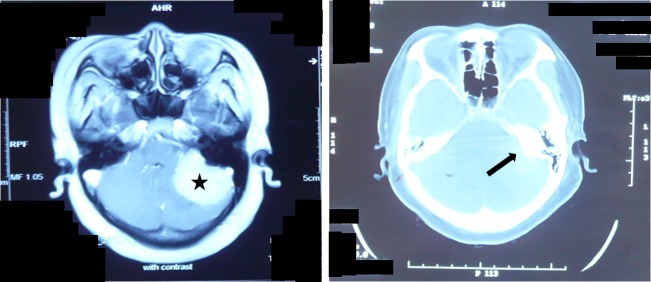
Reactive Thickening of the Petrous Bone MRI with contrast and CT demonstrating the location of the CPA meningioma and its petrous reactive changes in a 46-year-old female The CPA meningioma (star, left) was associated with a thickened petrous bone (arrow, right), but no dilated IAM. CPA: cerebellopontine angle; CT: computerized tomography; IAM: internal acoustic meatus; MRI: magnetic resonance imaging

Twenty-eight cases had tumors with reactive thickening of the petrous bone without dilated IAM (Figure 3).

Tumor resection

**Figure 4:**
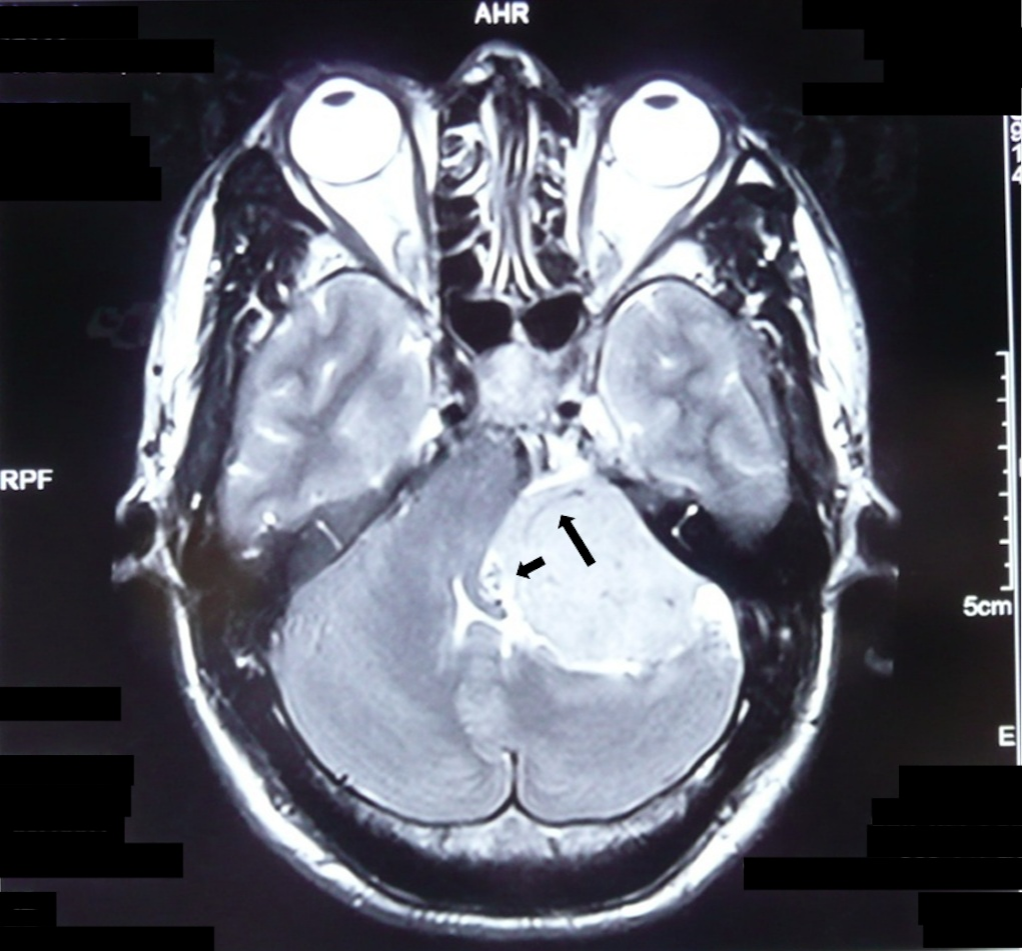
Feeding Vessels MRI with contrast demonstrating the blood supply to the meningioma in a 36-year-old male. This CPA meningioma had many adjacent vessels (short arrow) and perforating vessels (long arrow). CPA: cerebellopontine angle; MRI: magnetic resonance imaging

**Figure 5:**
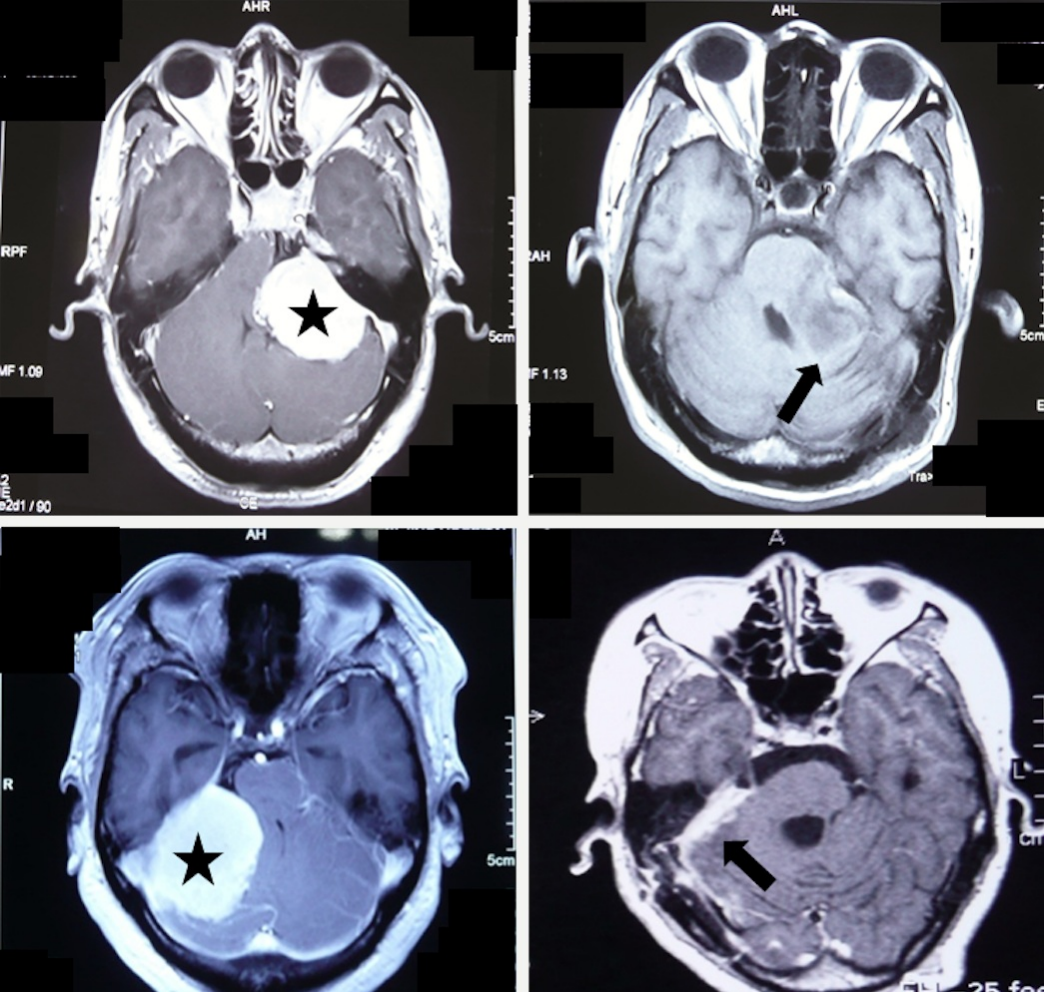
Total and Subtotal Resections MRI with contrast demonstrating the surgical removal of a CPA meningioma from two patients via a retrosigmoid approach. Upper row: a 36-year-old male who had total removal of his tumor (star). Lower row: a 48-year-old female with near total removal of her tumor (star). A small residual was left at the tumor origin at the back surface of the petrous bone. CPA: cerebellopontine angle; MRI: magnetic resonance imaging

**Figure 6:**
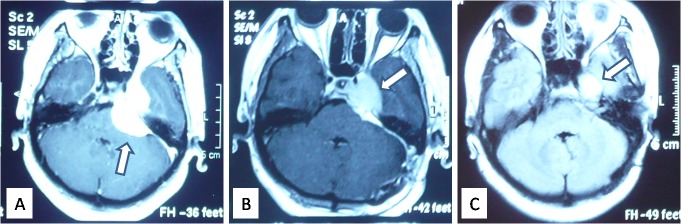
Partial Resection Contrast MRI demonstrating radiosurgery for residual from a CPA meningioma in a 47-year-old female presenting with left oculomotor palsy. Preoperative images revealed a straddle meningioma across the petrous apex (A, arrow). Partial removal of the tumor was achieved via a retrosigmoid approach. Residual tumor at its invasion of the cavernous sinus (B, arrow) was treated with Gamma Knife stereotactic radiosurgery (16 Gy) a month post-operation. Contrast MRI two years later showed obvious shrinkage of the residual tumor (C, arrow). CPA: cerebellopontine angle; MRI: magnetic resonance imaging

The adjacent and perforating vessels of the CPA meningiomas were well exposed and controlled (Figure 4). Thus, the blood supply to the tumors was greatly reduced. At areas of dura invasion, dural coagulation and removal were routinely done; reactive bone removal was also performed when it could be done safely. The degree of tumor resection was grossly categorized as total, subtotal (≥ 90%), or partial (< 90%). Total removal was achieved in 41 patients (83.7%) (Figure 5, upper row), subtotal in six patients (12.2%) (Figure 5, lower row), and partial in two patients (4.1%) (Figure 6).

Electrophysiological monitoring and cranial nerve protection

**Figure 7:**
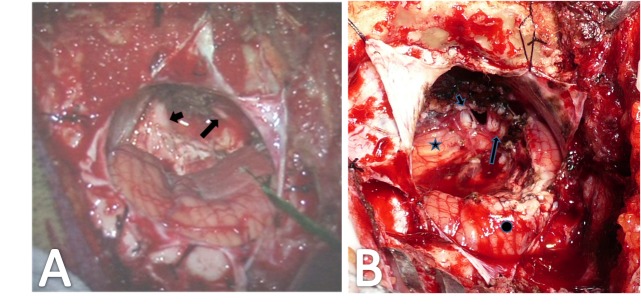
Intraoperative Exposure of Cranial Nerves Photograph of the intraoperative demonstration of anatomical preservation of the cranial nerves. (A) The left CPA meningioma was totally removed and the trigeminal (short arrow) and abducens (long arrow) nerves were anatomically preserved. (B) The trigeminal (short arrow) and facial/vestibulocochlear nerves (long arrow) were anatomically preserved following total removal of a right CPA meningioma. The brainstem (star) and cerebellum (round) were clearly seen. CPA: cerebellopontine angle

Neuroelectrophysiological monitoring (Epoch XP Neurological Workstation, Axon Systems, Hauppauge, NY) of the cranial nerves and brainstem functions was performed in 40 cases where the tumors were of close locations to the CNs. There were 21 patients who had abnormal electrophysiological changes to CN V, 29 patients to CN VII, and five patients to CN XI. Anatomical preservation of the trigeminal nerve (CN V) was achieved in 47 patients, accounting for 95.9% of the total cases (Figure 7A, B, short arrow). The abducens nerve (CN VI) was not monitored in this study. In one case, this nerve was preserved anatomically but lost its function postoperatively (Figure 7A, long arrow). The facial nerve (CN VII) was anatomically preserved in 44 cases (Figure 7B, long arrow), accounting for 89.8% of the whole series with CPA meningiomas. Of nine patients without neuromonitoring, two had ipsilateral facial numbness (trigeminal disturbance), three had facial nerve palsy, five had hearing deterioration, and one had dysphagia and cough weakness. Though not totally disappeared, these symptoms resolved to different degrees after discharge (within three weeks following admission).

Histological examination

All 49 cases (Figure 1) were diagnosed pathologically by WHO classification criteria as CPA meningioma; 47 were Grade I; the other two were Grade II. Local active growth was seen in the meningiomas by immunohistochemistry among five patients with Ki-67 staining of 20% of the tumor tissue (n = 1), 10% of the tumor tissue (n = 2), and 7% of the tumor tissue (n = 2). The tumor Ki-67 levels in all other cases were below 5%. The morphological subtypes of the meningiomas were meningotheliomatous (29 cases, Grade I), fibrous (12 cases, Grade I), transitional (three cases, Grade I), angiomatous (one case, Grade I), syncytial (one case, Grade I), clear (one case, Grade II), atypical (one case, Grade II), and mixed (one case, Grade I).

Immediate and delayed postoperative reactions

During the early postoperative period, nine patients had ipsilateral facial numbness (trigeminal disturbance), 12 had facial nerve palsy (24.5%), 29 had hearing deterioration or loss (59.2%), and 10 had dysphagia and cough weakness (20.4%). Though not totally disappeared, these symptoms resolved to different degrees after discharge (10 to 20 days following admission). In this series, one death occurred due to aspiration pneumonia, which might be the result of functional deterioration of either CN IX, CN X, or CN XII. Thus, the mortality rate of all was 2.0% (1/53). No case of CSF leakage was observed in this series.

Of the 12 who had facial nerve palsy, five had House-Brackmann (HB) Grade 2 facial nerve palsy and recovered to Grade 1 (normal) within 20 days. Four had HB Grade 3 facial nerve palsy and recovered to Grade 2 within three weeks and recovered to Grade 1 at the three to six-month follow-up. Three patients had HB Grade 4-5 facial nerve dysfunction and recovered to Grade 3 at the six-month follow-up.

Additional treatments

**Figure 8:**
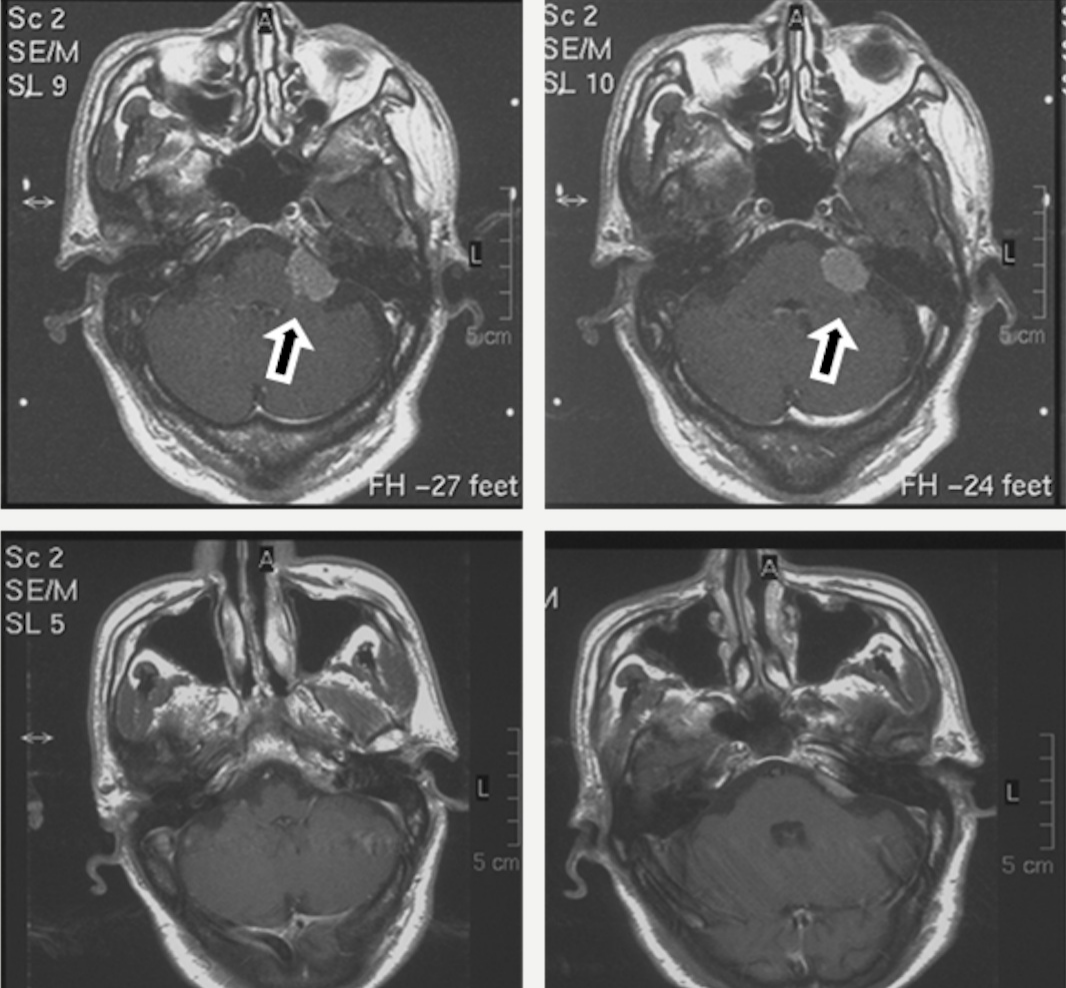
Application of Radiosurgery to Residual Tumor MRI with contrast of postoperative radiotherapy for the residual tumor. The residual of the CPA meningioma (upper row, arrow) underwent 16 Gy stereotactic radiosurgery 30 days after operation, and it disappeared two years later (lower row). CPA: cerebellopontine angle; MRI: magnetic resonance imaging

In this clinical series, Leksell Gamma Knife stereotactic radiosurgery was used as an important adjunct for the purpose of reducing postoperative recurrences and morbidities. This adjunct therapy was used in patients who had tumor invasion of the bone or extended dura, five patients who had an intraoperative residual tumor < 1.5 cm in diameter, and in 27 patients after total removal (Figure 8). It was started in a period between 20 to 180 days postoperatively.

Follow-up

Despite the aforementioned single mortality, 52 patients were successfully discharged. Forty patients (81.6% of the whole series) achieved postoperative follow-up from six to 42 months. The four cases treated with radiation were followed up for 25 to 34 months. Detailed follow-up was accomplished in 39 cases with a duration of three to 34 months (average: 24.5 months). The follow-up demonstrated a 98.1% survival rate (51/52) with one patient deceased from a non-operative cause (traffic accident). Three cases complained of facial numbness, five had mild hemifacial paralysis (HB Grade 2), 18 had hearing deterioration, two had hearing loss, and four had mild dysfunction of CN IX, CN X, and CN XI. Two cases with tumor pathologically classified as CPA meningioma WHO Grade II (clear and atypical) radiographically recurred, respectively, at the two and 2.5-year follow-ups despite early postoperative Gamma Knife intervention. One other patient with a pathological diagnosis of mixed tumor (WHO Grade I), who had no postoperative radiation, had a tumor recurrence 2.6 years later with the presentations of headache and gait instability. The other patients with small residuals had no tumor regrowth at the 34-month follow-up. Based on the high progression-free survival rate (approaching 100%), the Kaplan-Meier curve was not generated.

## Discussion

Analysis of the clinical presentations of the CPA meningiomas

CPA meningiomas often present with complicated symptoms. Common early symptoms, including hearing loss/tinnitus, headache, and vertigo, are often neglected by the patients. Inquiring about the early symptoms is crucial for evaluating the tumor origin. In this series, with an average duration of 19.4 months from the onset of initial symptoms to admission, early symptoms included hearing loss/tinnitus, vertigo, headache, gait instability, extremity numbness/weakness, facial pain/numbness, nausea/vomiting, facial palsy, hemifacial spasm, throat discomfort, swallowing disorder, and ptosis (oculomotor palsy). Among these, hearing disturbance, vertigo, and headache were the three most commonly seen complaints with occurrences of 42.9%, 40.8%, and 38.8%, respectively. A previous study reported that patients with CPA meningioma mainly complained of unsteadiness (54%), hearing loss (52%), vertigo (35%), and tinnitus (23%) [5]. Decreased hearing, vestibular signs, or impairment of corneal reflexes were the most common findings in the initial physical examinations. Signs of trigeminal nerve deficits were strongly correlated with the intrusion of the meningioma into the cavernous sinus or cerebellar tentorium. The symptoms presented by these patients depended primarily upon the tumor’s location, size, growth rate, and extension. Careful observation and examination helped to obtain an early diagnosis. There was one patient who initially suffered from headache and vertigo with the later onset of paroxysmal facial pain, which suggested the possibility of secondary involvement of the trigeminal nerve root (Figure 6). In our series, CN V, CN VII, and CN VIII were most frequently affected by tumors, followed by the oculomotor nerve (CN III). The posterior group of cranial nerves (CN IX, CN X, CN XI, and CN XII) were also involved in a few cases (one case with ptosis of the eyelid, one with throat discomfort, and one with a swallowing disorder). These cases challenged neurosurgeons with both bisecting and preserving the inflicted cranial nerves. Furthermore, there was a small portion of patients who, in spite of their larger tumors indicated by radiographic studies, presented with no obvious symptoms (4/49). Hence, advanced radiographic studies are necessary and important in making an early diagnosis.

Surgical strategy options

Because of the small sizes at early stages and slow growth, CPA meningiomas usually cause no apparent symptoms or neurologic deficits. Within this population, non-surgical interventions instead of radical operations should be first considered. In our group, four cases with the tumor size < 2 cm were treated with Gamma Knife stereotactic radiotherapy. They developed no indications for advanced surgical intervention in the follow-up periods (up to 25-34 months). MRIs revealed evidence of tumor shrinkage in some three out of the four cases (Figure 6, Figure 8). Clinical conditions, symptoms, signs, and tumor sizes are among the vital factors to be considered when choosing between surgical and conservative strategies.

We want to emphasize that the retrosigmoid approach used in our series is perhaps one of the most commonly used surgical approaches for removing CPA meningiomas. However, in deciding which surgical approach is the best, considerations need to be given to tumor invasion of the cavernous sinus, to their expansion to the IAM, to their tentorial attachment, and to their forward expansion towards the jugular foramen [5-6]. The consideration of the arachnoidal plane and the incorporation of arterial branches are also extremely important factors influencing the best surgical strategy and can significantly impact surgical outcome.

Although the meningiomas in this series were of various pathological subtypes and different sensitivities to radiation, subtotal or partial tumor removal for the protection of the cranial nerves and brainstem was possible. Jeopardizing a patient’s quality of life for the sake of total removal is not worthwhile. A small residual tumor could be well controlled via conservative treatments, such as radiotherapy. However, it should be emphasized that CPA meningiomas with WHO Grade II or higher levels of morphological evidence have a higher risk of recurrence in a relatively shorter period of time. Gross total resection is extremely important for these patients, and postoperative radiation should not be neglected.

Among the three patients who developed recurring tumors after the operation, two were confirmed pathologically as WHO Grade II (subtypes: clear, atypical) and one was a WHO Grade I (mixed subtype). Even though the former two patients received stereotactic radiation treatment early postoperatively, the tumors recurred in less than three years in accordance with the strong biological activities. Hardesty, et al. [7] did a retrospective study on 228 patients with microsurgically treated atypical meningiomas. Among them, 32 patients underwent stereotactic radiation therapy and had a recurrent rate of 25%, which is much lower than our's at 100% (2/2). The main reason for the discordance could be the small sample size in our study. The authors’ experience favors that cases with meningiomas of WHO Grade II or higher levels or a mixed type (WHO Grade I) be closely followed up with periodic radiographic studies. Since radiosurgery does not guarantee the prevention of the recurrence of tumors, complete surgical resection is still the main strategy to prevent tumor recurrence.

The surgeon’s choice of surgical approach depends mainly on the characteristics of the tumor, and his/her personal preferences. In this series, the retrosigmoid approach was chosen based on our understanding of the following parameters: tumor origin, growth direction, form and size, and the main symptoms and syndromes indicative of the invaded vital structures. Most neurosurgeons prefer a single approach, while others use certain combinations of the following approaches: retrosigmoid, transpetrosal, and transcochlear approaches. Leonetti, et al. reported a series of 29 patients with large CPA meningiomas (> 3 cm) treated surgically using a combined retrosigmoid-transpetrosal-transcochlear approach [8]. This combined lateral transtemporal approach provided wide exposure to the CPA and optimized the surgical extirpation. Some other authors suggest that the retrosigmoid approach has limitations, such as a restricted angle of view, high venous pressures, and suboptimal brain relaxation. They retrospectively evaluated a modified far-lateral approach in a consecutive series of 12 patients with large or giant posterior fossa convexity and CPA meningiomas. This approach incorporates transverse-sigmoid sinus exposure and C-1 laminectomy without condylar resection. Their evaluation showed that this modified far-lateral approach allows for a greater field of view, minimal venous bleeding, immediate access to the spinal subarachnoid space, and therefore, safe, and often total resection, of CPA meningiomas [9].

Surgical techniques

Generally, CPA meningiomas grow along the posterior aspect of the petrous part of the temporal bone in various directions and even extend into the cavernous sinus and the middle cranial fossa. However, the interface to which the tumor tightly adheres was often observed at the posterior surface of the petrous bone (from the tip to the root) in 85.7% of our cases, at the internal acoustic meatus in 6.1% of cases, and at the tentorium in 8.2% of cases. The fact that the majority of CPA meningiomas have originated from the posterior surface of the petrous bone provided a valuable clue for surgical separation. The first step for this kind of operation was to cut the blood supply to the tumor at its base using a bipolar electric coagulator, thus making it easier to separate the tumor from its base. After the tumor base—the most vascularized attachment (MVA)—had been well controlled, it was easier to separate the tumor body from the surrounding tissues because of its relatively loose connections therein.

Although difficult, there are many ways to distinguish the MVA from other contact surfaces. Based on operative findings from 34 consecutive patients with CPA meningiomas, Kunii, et al. [10] classified these tumors into four types: petroclival, in which the trigeminal nerve is displaced laterally; tentorial, in which the center of the tumor attachment is located at the medial tentorium; anterior petrous, in which the center of the tumor attachment is located anterior to the meatus; and posterior petrous, in which the center of the tumor attachment is located posterior to the meatus. Magnetic resonance imaging (MRI) was sufficient to confirm the attachment of the posterior petrous type. After reviewing the preop MRI films with our interventional neuroradiologists, the tumors deemed to have a rich vascular supply by both the neurosurgeon and neuroradiologists underwent angiography with possible embolization. In our series, 26 cases were analyzed angiographically and classified into three types: abnormal ipsilateral tentorial artery (type A); bilateral internal carotid artery (ICA) (type B); and nontentorial, non-ICA (type N). This angiographic classification was found to be useful in predicting meningioma attachments at the CPA. The existence of an abnormally developed tentorial artery was highly indicative of tumor attachment to the tentorium. A more sensitive method had been investigated to reveal the vascular supply to the meningioma. Yoshino, et al. reported that high spatial resolution three-dimensional computer graphics (hs-3DCG) was of a sufficiently high quality to enable a virtual operation field of the CPA meningioma [11]. This method may facilitate estimation of the MVA and the main feeder penetration point and help to choose the most appropriate approach to block the main feeder.

In order to reduce retraction injuries, a “piece by piece” method for removing the tumor under an operative microscope should be employed. The strategy requires that the tumor tissues be coagulated and even carbonized in advance, with an aim of obtaining tumor shrinkage and then bloodless resection. A cavitron ultrasonic surgical aspirator (CUSA®) (Integra LifeSciences Corp., Cincinnati, OH) was convenient for removing deep parts of CPA meningiomas. In our group of 49 cases, total removal was achieved in 41 (83.7%), subtotal in six (12.2%), and partial in two (4.1%). In another study, total removal was reported in 19 of 29 patients (67%) [8].

Preservation of cranial nerves

CPA meningiomas are usually found to arise from cells lining the arachnoid villi, which are occasionally observed close to nerve roots and their foramina, such as the IAM, compared with their predominant location adjacent to the dural sinuses. In our experiences, although the meningiomas tend to more loosely adhere to these often-inflicted cranial nerves (CN V, CN VII, and CN VIII) than acoustic neuromas are, separating them from cranial nerves is still challenging, especially when the tumor is located close to the foramina where the nerves enter or exit.

The trigeminal, facial, acoustic, and accessory nerves were among those usually monitored during operations. Considering the slow growth of meningiomas and for the purpose of protecting and preserving the cranial nerves to the best of our abilities, we left a small part of the tumors untouched (as a residual) in some cases, which were later treated with additional managements (i.e., Leksell Gamma Knife stereotactic radiosurgery) or staged operations. In the present group with intraoperative monitoring by neuroelectrophysiological techniques, 93.9% of cases (46/49) had anatomical preservation of the inflicted CN V, CN VII, and CN VIII. Although postoperatively, the functional status of these nerves initially revealed a disturbance to different degrees, each gradually recovered to a satisfactory level in 10 or more days. One patient (aged 64) suffered a severe disturbance postoperatively in the function of his posterior CN’s and later died of pulmonary inflammation. The part of the CPA meningioma compressing and adhering to the posterior CNs should be intentionally left behind to keep the nerves’ functions intact. Postoperative stereotactic radiosurgery can be a good remedial measure. Kane, et al. adopted a retrosigmoid approach for 22 cases with CPA meningioma and observed that 13 had an internal auditory canal (IAC) extension [5]. IAC drilling was needed to achieve a complete resection in only five patients; altogether 22 (91%) had improved or stable hearing postoperatively and one patient had permanent facial paralysis. CN’s IX and X had the most common complications (17% and 33%, respectively) and were almost exclusively associated with resection of tumor extensions into the jugular foramen (p < 0.01). The author concluded that CPA meningiomas could be removed with excellent rates of hearing and facial nerve preservation. Cautions must be exercised when attempting to resect tumors extending into the jugular foramen given the high rates of lower CN complications [5].

Neuroelectrophysiological monitoring

In the majority of cases, because of various tumor extensions, several cranial nerves (CN V through CN XI) were at risk of being touched, shifted, or even compressed, potentially leading to a variety of symptoms and syndromes. The facial nerve was most frequently involved in surgical complications, followed by the trigeminal and accessory nerves. In many instances, the facial nerve was torn in order to partially or completely disconnect the tumor from its surroundings. A small residual was permitted for protecting the functions of the CNs and brainstem. Intraoperative neuroelectrophysiological monitoring provided neurosurgeons with ongoing feedback regarding the cranial nerves. Warning signals from neuroelectrophysiological monitoring were immediately heeded since even anatomical preservation of the CNs did not guarantee intact functions. Due to certain practical problems, not all CN’s were monitored intraoperatively. In one case of this series, the abducens nerve was clearly exposed and confirmed to be intact after being separated from the tumor but ended up functionally impaired postoperatively (Figure 7A). In this series, the rate of anatomical preservation of the facial nerve was 93.9%; nevertheless, facial nerve palsy occurred in 24.4% of cases. Separation of the facial nerve from the tumor might impair the facial nerve’s functions. For these cases, neuroprotective and microcirculation-improving drugs, as well as hyperbaric oxygen therapy, are necessary for later treatment and functional rehabilitation.

Remedial measures

Because of the possible complications following surgical manipulation, cautions should be exercised when choosing to operate on a tumor of small size or to perform a complete removal of a small-sized residual. The difficulty and danger arise during surgery of a tumor involving the dura anterior to the IAM, in comparison with the treatment of the part posterior to the IAM [3]. Under the former circumstance, compromise should be made to keep intact the important structures. Gamma Knife stereotactic radiosurgery should be considered as an important adjunct for the purpose of reducing postoperative reactions and morbidities. In this study, stereotactic radiosurgery was used to treat the patients who had a small CPA meningioma with a diameter < 2 cm without surgical interventions or who had an intraoperative residual which was < 1.5 cm in diameter. Small tumors were thought to be more sensitive to radiation dose given by stereotactic radiosurgery [12]. A radiation dose of 12 - 15 Gy was used in this group [4].

The meningotheliomatous meningioma was the main subtype in this series, dominating 59.2% (29/49) of the whole group. Another 22.5% (12/49) had a fibrous subtype. The other eight cases (< 16.3% of the total operative patients) exhibited transitional (3/49), angiomatous (1/49), syncytial (1/49), mixed (1/49), clear (1/49), and atypical (1/49) subtypes. Our follow-up suggested that there was a greater tendency for recurrence in less than three years with subtypes of clear, atypical, and mixed, even though two Grade II patients received postoperative Gamma Knife stereotactic radiosurgical intervention early. The recurrence rate in our series was 6.1% (3/49). The clear, atypical subtypes are classified as WHO Grade II, and the mixed type is classified as WHO Grade I. Hence, the biological characteristics were in accordance with the recurrence. Clear cell meningiomas (CCM), a rare histological subtype of meningioma, have a high recurrence rate and aggressiveness. A study with a series of 15 patients harboring intracranial CCM (mainly located at the CPA) demonstrated that immunohistochemistry played a vital role in differentiating CCM from other tumors. Brain invasion, atypia, and MIB-1 labeling index were likely to predict the recurrence. The extent of resection could be associated with the prognosis as well [13]. An extremely unusual case of cystic angiomatous meningioma in the CPA region was reported in a 58-year-old male patient. Histopathology revealed an angiomatous meningioma with a predominant microvascular component and extensive cystic changes [14].

In one case, stereotactic radiosurgery was reported to be effective for treating CPA meningioma [15]. An older adult female with a large bilateral CPA meningioma, who presented with tinnitus and dizziness but nearly normal hearing, was treated with Gamma Knife intervention at a dose of 14 Gy on each side with an interval of one year between the two radiation treatments. The follow-up radiographic studies revealed that the tumors did not progress in six years from the first radiosurgery; her hearing and bilateral facial function also remained intact and normal. This report indicates that preservation of CN function was generally possible after stereotactic radiosurgery for CPA meningioma [15]. Therefore, we strongly advise that stereotactic radiosurgery be considered as the first option for patients who have multiple comorbidities (i.e., those who are too weak to undergo operation), small-sized tumors (with diameters < 2 cm), a residual (left behind as a compromise for reducing intraoperative injury), and bilateral tumors (with no obvious auditory and facial symptoms).

Reinert, et al. analyzed a total of 201 operated cases with an intracranial meningioma with diameters ≤ 3 cm. According to tumor locations, patients were classified as group I (cranial vault, parasagittal, and lateral sphenoid), group II (falx, frontobasal, medial sphenoid, parasellar, and tentorial), and group III (cavernous sinus, petroclival, petrosal, CPA, and foramen magnum) [16]. Radical removal was achieved in 100% in group I, in 93.7% of group II, and in 80% of group III. No disease-related mortality was observed. Microsurgery provides excellent efficacy and morbidity results for patients with group I and II meningiomas, especially in asymptomatic patients; therefore, it may be considered the first choice of treatment for these patients. The results of microsurgery in group III were worse than those of stereotactic radiosurgery reported in the literature.

Limitations

We recognize that this study is limited by the inherent problem of a retrospective analysis. In addition, every surgeon's experience and technique skills vary, influencing the outcomes of the patients.

## Conclusions

In this study, we reviewed a large sample size of 53 patients with CPA meningioma. Though generally considered as a big challenge to resect tumors at this location, retrosigmoid approach yielded satisfactory results with an excellent extension of resection, preservation of cranial nerves, and low complication rates in our institution. Meanwhile, stereotactic radiation therapy can be used as an adjunct for small or high-grade tumors. Thus, we advocate this approach for CPA meningiomas that are surgically resectable.
